# Recent advances in machine learning and coarse-grained potentials for biomolecular simulations

**DOI:** 10.1016/j.bpj.2025.06.019

**Published:** 2025-06-20

**Authors:** Adolfo B. Poma, Alejandra Hinostroza Caldas, Luis F. Cofas-Vargas, Michael S. Jones, Andrew L. Ferguson, Leonardo Medrano Sandonas

**Affiliations:** 1 Department of Biosystems and Soft Matter, Institute of Fundamental Technological Research, Polish Academy of Sciences, ul. Pawińskiego 5B, 02-106 Warsaw, Poland; 2 Universidad Nacional de Ingeniería, Av. Túpac Amaru 210, Rímac, Lima 15333, Peru; 3 Pritzker School of Molecular Engineering, University of Chicago, Chicago, Illinois; 4 Department of Chemistry, University of Chicago, Chicago, Illinois; 5 Institute for Materials Science and Max Bergmann Center of Biomaterials, TUD Dresden University of Technology, 01062 Dresden, Germany

## Abstract

Biomolecular simulations played a crucial role in advancing our understanding of the complex dynamics in biological systems with applications ranging from drug discovery to the molecular characterization of virus-host interactions. Despite their success, biomolecular simulations face inherent challenges due to the multiscale nature of biological processes, which involve intricate interactions across a wide range of length scales and timescales. All-atom (AA) molecular dynamics provides detailed insights at atomistic resolution, yet it remains limited by computational constraints, capturing only short timescales and small conformational changes. In contrast, coarse-grained (CG) models extend simulations to biologically relevant time and length scales by reducing molecular complexity. However, CG models sacrifice atomic-level accuracy, making the parameterization of reliable and transferable potentials a persistent challenge. This review discusses recent advancements in machine learning (ML)-driven biomolecular simulations, including the development of ML potentials with quantum-mechanical accuracy, ML-assisted backmapping strategies from CG to AA resolutions, and widely used CG potentials. By integrating ML and CG approaches, researchers can enhance simulation accuracy while extending time and length scales, overcoming key limitations in the study of biomolecular systems.

## Significance

Machine learning (ML) has emerged as a transformative tool for bridging the gap between high-resolution all-atom models—which explicitly represent every atom in a molecular system—and low-resolution coarse-grained approaches, which reduce complexity by grouping atoms into larger interaction units. On one hand, ML potentials trained on quantum-mechanical data enable accurate energy predictions with high transferability. On the other hand, ML also enhances coarse-grained models by improving structure reconstruction through advanced backmapping strategies.

## Introduction

Biomolecular simulations have enriched our physical and chemical understanding of the structure-function relationship in biological systems, from single molecules ranging from proteins to large molecular assemblies in crowded cellular environments (1, 2). In many cases, such knowledge has found applications in medical and life sciences overcoming length scale and timescale limitations such as the characterization of physicochemical properties of HIV-1 capsid (3), role of glycans in the activation of severe acute respiratory syndrome coronavirus 2 (SARS-CoV-2) spike protein for cell recognition (4), the discovery of cryptic pockets in protein (5, 6), and unveiling the mechanical stability of pathogen adhesin (7). An essential feature of these systems is their multicomponent nature and the interplay of different length scales and timescales in the emergence of complexity.

The timescales of biological processes, such as protein folding, protein recognition, and transitions between different metastable states, among others, range from $10^{-6}$ to $10^{-3}$ s, although certain processes, especially those involving larger proteins or more complex systems, can extend to seconds or longer. Thus, they are orders of magnitude slower than typical molecular motions of interest (e.g., backbone fluctuations, side-chain rotations) that typically occur on a nanosecond to microsecond timescale (i.e., $10^{-9}-10^{-6}$ s). The length scales of molecular rearrangements are also much smaller in all-atom molecular dynamics (AA-MD) simulation than those of larger structural changes typically observed experimentally in biological systems. Moreover, parameterizing accurate and transferable all-atom potentials remains a long-standing challenge in biomolecular simulations. On one hand, classical force fields such as AMBER, CHARMM, and OPLS rely on a set of physically motivated mathematical functions to describe intra- and intermolecular interactions. However, applying these models across diverse and complex molecular systems often requires system-specific reparameterization and extensive fine-tuning to achieve the desired accuracy in predicting structural and thermodynamic properties. On the other hand, quantum-mechanical (QM) methods (e.g., coupled cluster, density functional theory) offer the ability to accurately describe molecular interactions across a wide variety of systems. However, they remain computationally prohibitive for larger systems (greater than thousands of atoms) and long timescales, even with the most efficient algorithms and state-of-the-art supercomputers (8, 9). This presents a barrier to the application of QM methods for addressing biomolecular problems, such as protein mutations or structural transitions. To mitigate this issue, QM methods have been integrated with machine learning (ML) techniques, leading to the development of well-established approaches aimed at generating efficient, accurate, scalable, and transferable (EAST) (10, 11) ML models to advance biomolecular simulations with atomistic resolution. Among them, we have ML models that approximate the potential energy surface (PES) of molecular systems using state-of-the-art neural network architectures trained on QM property data (commonly referred to as machine-learned potentials, MLPs) (12,13,14,15,16,17,18,19,20,21), the augmentation of semiempirical methods with ML techniques (22,23,24,25,26,27,28,29), and the development of hybrid molecular mechanics/ML approaches (30,31,32,33,34,35).

Despite their precision, MLPs still face challenges related to computational cost relative to classical force fields, which can limit their application to smaller systems or shorter simulation timescales of only a few nanoseconds. Accessing large conformational changes in proteins on the scale of thousands of nanoseconds is still not possible with MLPs. In this regard, pragmatic coarse-grained (CG) approaches (36,37,38,39) enable the exploration of large time and length scales while preserving a molecular-level representation of the studied systems. Several CG potentials have been instrumental in studying large-scale processes such as conformational changes in proteins and the assembly of macromolecular complexes (40, 41) over extended scales such as chromatin organization (≈ 150–250 nm) (42), the nanomechanics of microtubes (1–12 *μ*m) on the millisecond timescale (43), and the ensemble conformations of intrinsically disordered proteins (IDPs) and multidomain proteins (44). Among these potentials, the CALVADOS CG potential (45) employs Bayesian optimization of the hydrophobicity scale for each amino acid residue, with interaction sites optimized to reproduce ensemble conformations accurately. Moreover, ML approaches based on neural networks have been recently applied to define CG potentials for proteins. CGnets (46, 47) and its extended version (48) were developed to reproduce conformations aligned with the free energy landscape, and Boltzmann Generators (49) learned transformations for efficient sampling of equilibrium states. These methods demonstrate strong progress in modeling transitions between metastable states and pave the way for their extension to more complex systems such as RNA-protein or protein-ligand assemblies. Building on these complementary advances, researchers are now integrating the strengths of ML and CG approaches to develop hybrid methodologies that balance accuracy with computational efficiency. ML techniques can help parametrize CG models (46), generate CG structures and backmap them to higher resolutions (50), define optimal parameters and the degree of CG resolution (45), evaluate the quality of CG mapping (51), and run CG simulations using ML potentials (48). This synergy holds the potential to overcome long-standing challenges in biomolecular simulations, enabling detailed interaction modeling while accessing extensive configurational landscapes.

**Figure 1:**
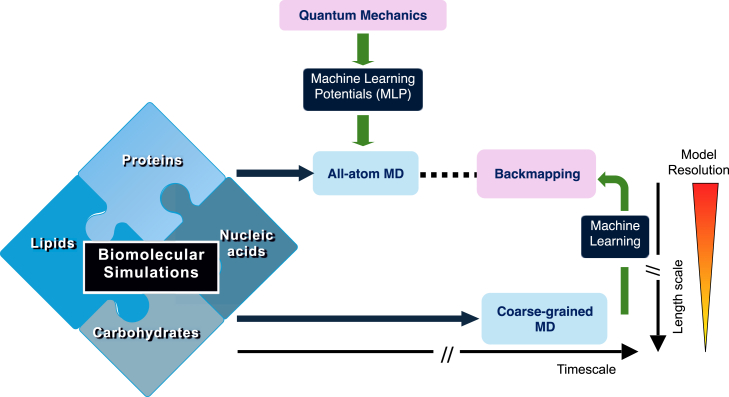
A cartoon illustrating the approaches for biomolecular simulations discussed in this review: (1) high-resolution models (all-atom MD), which are limited to small length scales and timescales but enhanced by machine learning potentials (MLPs), and (2) low-resolution models (coarse-grained MD), which enable significantly larger time and length scales, with backmapping to higher resolutions facilitated by ML. The dashed line represents the connection between enhanced all-atom MD (via MLP) and the backmapping results at long timescales, which are currently under development.

This short review explores recent strategies aimed at enhancing the reliability of biomolecular simulations. It is designed for a broad biophysical audience and provides an overview of key methodologies that advance biomolecular modeling toward larger length scales while maintaining high accuracy (see Fig. 1). Bhatia et al. (52) recently presented a review on ML for multiscale modeling and Gkeka et al. (53) on the integration of ML with MD more generally. We expand on topics from both of these reviews while focusing on particular themes in CG simulation. In our work, the first section discusses the development of QM-based ML potentials for biomolecules and emphasizes successful applications and limitations. The second section highlights several pragmatic CG potentials for multicomponent system (36, 54,55,56) and statistical CG potentials that have demonstrated versatility in describing biomolecular complexes (57). These include models for lipids, nucleic acids, polysaccharides, and their posttranslational modifications of proteins, which have gained increasing support from the biophysical and physicochemical communities. The third section examines ML-driven approaches for backmapping representative structures of the CG trajectories to higher-resolution atomistic representations. Finally, we conclude by discussing the immediate challenges facing ML potentials, CG potentials, and backmapping methods in their pursuit of more transferable, accurate, and thermodynamically consistent biomolecular potentials.

## Atomistic machine-learned potentials

### Pure quantum-mechanical ML potentials

**Figure 2:**
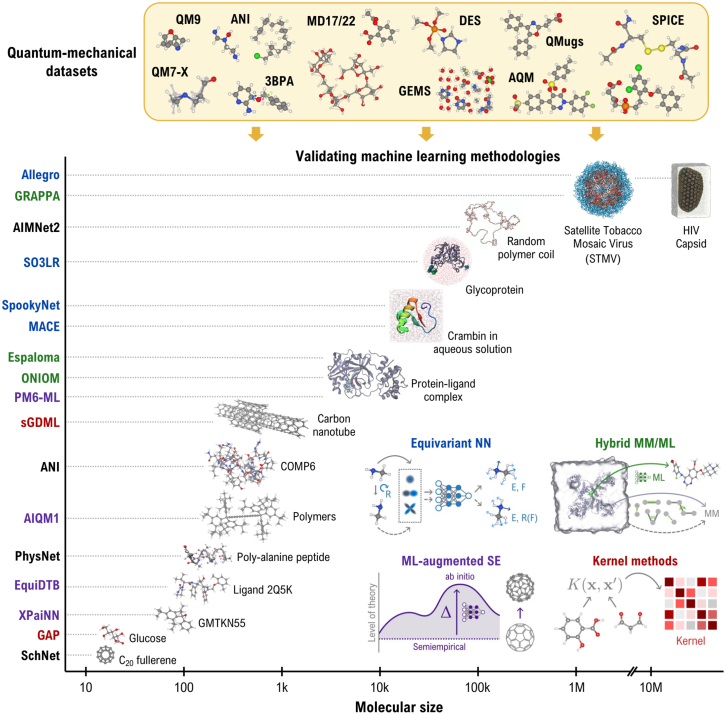
Evolution of the largest (bio)molecular systems investigated using machine learning (ML) potentials with quantum-mechanical (QM) accuracy. We highlight the key QM datasets employed in training these potentials. A schematic representation of the ML methodologies employed in related works is provided in the graph. The color coding of the ML potentials on the y-axis corresponds to the different ML methodologies. Figures of (bio)molecular systems have been adapted from the references corresponding to each ML methodology, except for Crambin (13), Glycoprotein (19), random polymer coil (60), Satellite Tobacco Mosaic Virus (35), and HIV capsid (3) systems, which were extracted from the respective references.

Building on groundbreaking methods like the high-dimensional Behler-Parrinello Neural Network (BPNN) (58) and Gaussian Approximation Potentials (GAP) (59), a diverse range of methodologies have been developed using kernel and deep learning methods to parameterize MLPs that meet EAST requirements for performing biomolecular simulations; see Fig. 2. Although BPNNs describe atomic environments using symmetry functions and element-specific NNs to compute atomic energies (58), GAP employs Gaussian process regression to model local atomic interactions, utilizing descriptors that map Cartesian atomic coordinates to invariant representations (59). The outstanding progress in MLPs has also been possible due to the increased availability of extensive QM datasets of small and large molecular complexes for training MLPs (e.g., QM7-X (61), ANI (62), QM9 (63), MD17/22 (64, 65), DES (66), GEMS (13), QMugs (67), Aquamarine (68), SPICE (69)). In this sense, kernel-based approaches such as GAP (70) and Gradient Domain Machine Learning (GDML) (64) have been successfully demonstrated to produce reliable MLPs for small molecules with reduced training sets and at QM accuracy. Unlike GAP, GDML learns conservative force fields directly from atomic force data, bypassing the need to fit energies explicitly. The incorporation of spatial and temporal physical symmetries into the GDML method, known as the symmetrized GDML model (71), along with the optimization of the training algorithm, enables the prediction of the PES of molecular systems containing up to a few hundred atoms, such as carbohydrates, nucleic acids, and supramolecules (65).

Although powerful and data-efficient, kernel-based approaches are less flexible and can only be used to develop potentials for single systems. An alternative that has advanced rapidly in recent years is the use of NNs for parameterizing MLPs. Accordingly, SchNet (72, 73) and ANI (62, 74) models were initially used to reconstruct the PES of small molecules at high levels of theory such as coupled cluster with single, double, and perturbative triple excitations method at the complete basis set limit (CCSD(T)/CBS) and density functional theory (DFT) using hybrid PBE0 functional supplemented with a many-body dispersion treatment (PBE0+MBD). The limitations in scalability and generalizability arising from the molecular representation and architecture design employed in both models motivated the development of more physically and chemically inspired NN approaches. For instance, the performance of the AIMNet model (Atoms-in-Molecules Neural Network) (75) relies on multitask learning to simultaneously predict molecular and atomic QM properties, including energies, atomic charges, and volumes. The second generation of this model, namely AIMNet2 (60), extends the chemical coverage to 14 atom types and augments the local configurational energy (AIM layer) with long-range electrostatics and dispersion interactions for investigating organic compounds with varied charges and valency. A similar methodology underpins PhysNet (76), which refines energy predictions by accounting for the physical models of electrostatics and van der Waals interactions (DFT-D3) (77) during the training procedure. This approach allows PhysNet to capture long-range interactions accurately, addressing the limitations presented in earlier models and extending the applicability of NN-based potentials to a broader range of molecular systems (e.g., polyalanine peptide, SN_2_ reactions).

More sophisticated NN architectures have recently been developed for constructing more generalizable MLPs by incorporating equivariance in the molecular representation. Indeed, building on PhysNet, the equivariant model SpookyNet (12) incorporates physically inspired modules that describe local and nonlocal pairwise interactions such as nuclear repulsion, electrostatics, and dispersion. Besides achieving state-of-the-art accuracy in energy and force prediction on benchmarks such as QM7-X (61) and MD17 (64), the SpookyNet model, trained on QM data of bottom-up and top-down molecular fragments (GEMS datasets), can accurately predict the structural properties of large and complex biomolecules such as polyalanine peptides, crambin in aqueous solution, and gas-phase binding curves of angiotensin-converting enzyme 2 and the receptor-binding domain of the SARS-CoV spike protein (13). Allegro (14) is another equivariant NN model that combines message passing NNs (MPNNs) with strict locality, effectively capturing local atomic interactions while maintaining a global view of the system. This model has proven transferable to high-temperature MD simulations and is scalable through GPU parallelization, enabling simulations with tens of millions of atoms (e.g., VIH capsid) (15). Using the local descriptors defined by the Allegro model, the FENNIX model (Force-Field-Enhanced Neural Network InteraXions) (16) was developed to predict short-range energy, atomic charges, and volume. Similar to SpookyNet, these atomic properties are integrated into physically motivated functional forms for calculating electrostatic and dispersion energy contributions, enabling the investigation of relevant simulations at QM accuracy, such as the torsional free energy profile of solvated alanine dipeptide and bond dissociation energy profiles.

Unlike previous models that explicitly use two-body interactions, the MACE (17) model is built on an equivariant MPNN, which leverages higher-order symmetric features for each atom. This property enables the MACE model to more effectively capture QM many-body effects in the PES of molecular systems. Indeed, the MACE-OFF23 model (18) was recently developed using diverse QM datasets comprising small and large single molecules as well as molecular dimers. This flexible model can calculate torsion profiles of drug-like molecules, enthalpies of molecular crystals, folding dynamics of alanine, and the power spectrum of crambin in explicit solvent. Following a similar reasoning, the SO3LR model (19) was recently developed using an updated version of the SO3krates (20, 21) equivariant NN architecture, which now incorporates both short- and long-range physical models in its design. The applicability of the SO3LR model was demonstrated through nanosecond-long simulations with QM accuracy across a broad chemical space, including small biomolecular units, polyalanine systems, bulk water, the crambin protein, an N-linked glycoprotein, and a lipid bilayer.

### ML-augmented semiempirical methods

Semiempirical (SE) methods strike a balance between classical potentials and DFT methods in terms of EAST requirements. As the simplest form of electronic structure theory, SE methods employ integral approximations and predefined functional forms of atomic interactions to accelerate calculations. To reduce errors, they incorporate parameterizations based on reliable experimental or theoretical reference data (78). Although this compromises their accuracy, SE methods remain highly efficient for large systems with $10^{2}$–$10^{4}$ atoms, where classical treatments would be inadequate due to complex QM effects. In this context, ML has the potential to enhance both the accuracy and transferability of SE methods while preserving their computational efficiency (79).

By leveraging the fact that SE methods already capture much of the relevant physics, the Δ-learning approach has emerged as a powerful strategy for enhancing their accuracy. In this approach, the target property is the difference between SE energies and forces and their DFT counterparts, enabling SE methods to achieve higher accuracy in physicochemical properties with minimal computational overhead. An early example of this methodology was demonstrated for a set of C_7_H_10_O_2_ isomers from the GDB17 data set (22, 23). Here, a KRR model was trained to predict atomization energies at the G4MP2 level (80) by learning the difference between G4MP2 and PM7 (81) semiempirical energies, outperforming models trained on absolute energies. Similarly, a BPNN has been used to address the limitations of the second-order density functional tight-binding (DFTB2) (82) method in describing intramolecular hydrogen bonds and torsional potentials of glycine (24). Building on this idea, the accuracy of the third-order DFTB method (83) was improved by replacing the standard pairwise repulsive potential with a many-body NN repulsive potential, NN_rep_ (25), trained using the SchNet architecture. This advancement achieved hybrid DFT-PBE0-level accuracy across energetic, structural, and vibrational properties of small molecules. To overcome the scalability and transferability limitations of NN_rep_, the EquiDTB framework was recently introduced (26). By leveraging physics-inspired equivariant NNs to parameterize many-body $\Delta_{\text{TB}}$ potentials, EquiDTB extends the applicability of this ML-corrected DFTB approach to larger molecules and noncovalent systems, surpassing the chemical space covered by the original training QM datasets.

Recent advancements in the use of Δ-learning for (bio)molecular simulations have exemplified its great potential to develop a more generalizable hybrid QM/ML computational method. For instance, the AIQM1 model (general-purpose artificial intelligence QM method 1) (27) was developed by targeting the energy differences between the orthogonalization- and dispersion-corrected method 2 Hamiltonian (ODM2) (84) and CCSD(T)^∗^/CBS (85) calculations from the ANI-1ccx data set (86), yielding “gold-standard” coupled-cluster accuracies for the ground-state properties, such as energies and geometries, of closed-shell, neutral organic compounds like polyynes, molecular dimers, and water clusters. Another notable Δ-learning model is PM6-ML (28), which integrates the robust PM6-D3H4X semiempirical method (87) with the TorchMD-NET architecture (88) to achieve DFT-level accuracy. PM6-ML covers a broad chemical space, ranging from small peptides to protein-binding complexes, and is capable of predicting interaction energies, conformational energies, torsional profiles, and optimized geometries. In a similar vein, the XPaiNN model (29) was developed to enhance the performance of GFN2-xTB (89) using the PaiNN architecture (90). Indeed, XPaiNN has demonstrated improved accuracy and transferability in investigating large conjugated compounds, noncovalent intermolecular interactions, and transition states.

### Hybrid MM/ML models

Molecular mechanics (MM) potentials represent molecular systems as atomic point masses interacting through physical models that describe bonded and nonbonded interatomic interactions. Although efficient and capable of handling long-timescale simulations, MM potentials have notable limitations in accurately describing QM effects in complex (bio)molecules. These limitations arise from geometrical and electronic constraints in the parameterization procedure, which affect their transferability and generalizability. To address these shortcomings, recent efforts have focused on using QM-based MLPs to simulate intramolecular interactions within the solute, whereas solvent-solvent and solvent-solute interactions are treated using MM potentials. For instance, the hybrid ANI-2x/AMEBA approach (30) was developed to achieve CCSD(T)-level accuracy by combining the ANI-2x potential for describing interatomic interactions in proteins and nucleic acids with the AMEBA polarizable potentials (91) for modeling interactions with the chemical environment. This hybrid model significantly improves the accuracy of solvation and absolute binding free energy calculations compared with using AMEBA alone (30).

Unlike the ANI-2x/AMEBA approach, which relies on a pretrained ML model, the hybrid ANA2B model (31) is the result of a fundamentally different training procedure that considers an anisotropic MPNN to estimate intramolecular interactions (92), and a fully connected NN to parameterize intermolecular short-range pairwise interactions. These short-range interactions are further complemented by D3 dispersion correction and other long-range intermolecular interactions, which are modeled using physically motivated terms derived from MM potentials. Although ANA2B is not explicitly trained on condensed-phase systems, it demonstrates strong transferability, making it a promising candidate for simulations in such environments. Building on this concept, the ONIOM approach (Our own N-layered Integrated molecular Orbital and molecular Mechanics) (32) combines MLPs at multiple levels of theory (e.g., coupled-cluster, DFT) with SE methods and MM potentials to describe interatomic interactions in different regions of a given biosystem. This multiscale strategy enables highly accurate quantum refinement of the crystallographic structures of diverse protein-drug/inhibitor systems with computational costs significantly lower than those of pure QM methods, paving the way for applications in molecular recognition, catalysis, and drug development.

On the other hand, hybrid MM/ML models like Espaloma (33) have replaced the discrete atom-typing scheme with continuous atomic representations generated by graph NNs to construct end-to-end optimizable force fields. These representations are trained on small molecules, peptides, and nucleic acids and are then mapped to MM parameters for atoms, bonds, angles, and torsions. This approach enhances chemical diversity, improves accuracy, and expands the applicability of MM potentials to more relevant biomolecular systems, e.g., folded proteins and protein-ligand complexes (34). The GRAPPA model (35) adopts a similar strategy but includes a graph attention NN, inspired by the transformer architecture, to map atom embeddings to MM parameters. This design improves expressivity by enforcing only the required permutation symmetries, providing greater flexibility in learning complex interactions and enabling superior performance compared with traditional MM potentials such as Amber99SB-ILDN (93) and Gaff-2.11 (94). Its capabilities were demonstrated through condensed-phase MD simulations of the large and complex Satellite Tobacco Mosaic Virus (35), which consists of a single-stranded RNA genome packaged inside a simple protein shell (capsid), totaling approximately one million atoms (95)—a suitable biomolecular system for benchmarking the computational efficiency of ML approaches.

## Coarse-grained potentials

### SIRAH

**Figure 3:**
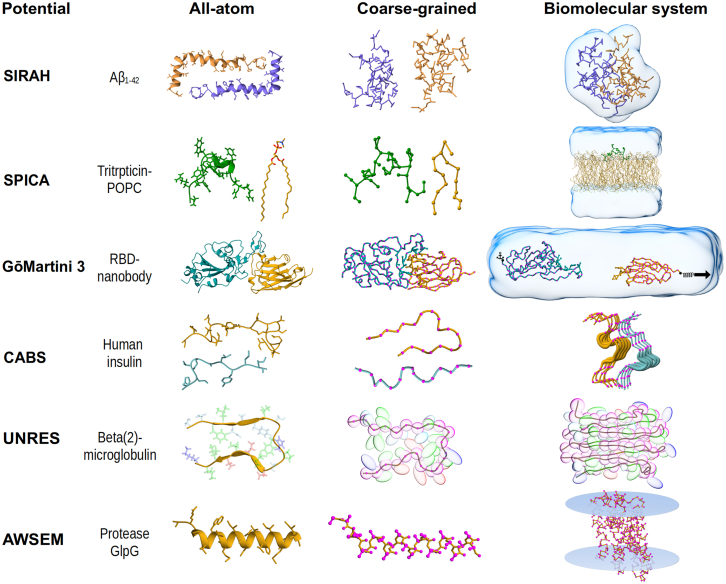
CG potentials and their applications in biomolecular systems. Each example shows the AA and CG representations, along with a protein complex in CG description. When present, solvent is depicted as a light blue transparent surface. Structures were visualized using ChimeraX (96) and are based on open-source PDB entries, which do not require special permission for use. Data sources for protein complex: SIRAH (55) (PDB ID: 1ITY), SPICA (97) (PDB ID: 1D6X), GōMartini 3 (98) (PDB ID: 6ZH9), CABS (57) (PDB ID: 3I40), UNRES (99) (PDB ID: 2E8D), and AWSEM (100) (PDB ID: 2XOV).

This CG potential (see Fig. 3) was introduced in 2010 (101) for the study of DNA systems. It was later expanded to protein systems (102), and in its last version, SIRAH 2.0 can describe metal ion coordination, unbiased conformational sampling for free energy calculations, and specific protein-peptide recognition (103, 104). This CG model was developed following a top-down approach that fits interactions to structural data from sources like the Protein Data Bank (PDB) and canonical structures such as B-DNA, *α*-helices, and *β*-peptides. It avoids using specific algorithms for parameter development, allowing flexibility through trial-and-error to enhance resolution in key areas. Unlike other CG potentials, SIRAH is unbiased, avoiding artificial constraints on secondary structures (see Table 1 and Fig. 3). Partial charges on each bead create effective dipole moments, allowing the CG model to mimic dielectric permittivity and tune ionic strength in simulations. This capability enables ion-specific effects on DNA that are consistent with observations from high-resolution structures. A study on the single-chain conformational ensembles of an IDP in SIRAH (105) showed good agreement with the nuclear magnetic resonance (NMR) conformers. However, reproducing single-chain properties is not sufficient for interpretation of the phase diagram in protein condensates (106), particularly when transient secondary protein structures (e.g., *β*, *α*, and coil) do not switch between themselves in CG simulations, which has yet to be captured. Those conformational changes are key for the stabilization of protein condensates transitioning to the aberrant phase (e.g., *β*-amyloid fibrils) and still face real challenges (107). Martins and Galamba recently employed SIRAH for the study of the monomer and small oligomers of *α*-synuclein (108). Due to the high resolution of the SIRAH protein backbone (i.e., N-C*α*-O), the model reproduced $R_{g}$ values in the range of ≈ 2 nm according to AA-MD simulations generated by Amber99sb and Charmm36m. However, SIRAH model has not yet been used to explore protein condensates, and careful interpretation should be done in case of capturing single-molecule properties of IDPs for prediction of biomolecular phase diagrams (106). Robles et al. (109) investigated the conformational changes associated with activation or inactivation by an agonist (p-TA) and antagonists (EPPTB and RTI) in the human trace amine-associated receptor (hTAAR1), which is a G-protein-coupled receptor (GPCR). Elucidating the mechanics of activation can play a role in the development of a treatment of neuropsychiatric condition. CG simulations confirmed in timescale of 5 *μ*s that G-protein interacts with intracellular loop 3 (ICL3) as well as with intracellular segments of several TMDs. Agonist binding promotes compact receptor conformations associated with activation, whereas antagonists stabilize the receptor in an inactive state. These findings illuminate the dynamic nature of GPCR modulation and the critical role of ICL3 movement in receptor signaling. The CG potentials, simulation tools, and parameters can be accessed freely from the webpage (https://www.sirahff.com/).

**Table 1:** Overview of CG potentials: amino acid resolution, parameters for capturing protein flexibility, and versatility in modeling biomolecular complexes

Potential	Resolution	Protein flexibility	Available biomolecules for complex formation
SIRAH	(3–8 beads)	Fair parametrization of secondary/tertiary structure.	Water, electrolytes, lipids, DNA, metal ions, and proteins with posttranslational modifications.
SPICA	(1–4 beads)	EN model is established between two CG beads when they are separated by more than two bonds or by a spatial distance of less than 0.9 nm; Force constant: 1.195 kcal/(Å^2^·mol).	Explicit solvent (water), lipids, ions, surfactants.
GōMartini 3	(1–6 beads)	Gō bonds mapped as LJ potentials with *ϵ* in the range 9.4–15.0 kJ/mol and $\sigma =d/2^{1/6}$, where *d* is the C*α*–C*α* distance from a native contact.	Explicit solvent (e.g., water, hexadecane), lipids, ions, nucleic acids, carbohydrates, small biomolecules, and more.
CABS	(2–3 beads)	Soft flat-bottom distance restraints (± 1 Å) between C*α* atoms.	Implicit solvent, proteins.
UNRES	(2 beads)	Not user-adjustable; based on physics-derived potentials and statistical knowledge.	Implicit solvent, proteins, DNA.
AWSEM	(2–3 beads)	Residue-specific burial potential, preserving packing and solvent accessibility; modeled with a sigmoidal-like function, tunable via $\lambda_{\text{burial}}\approx 0.3$–1.0.	Implicit solvent, proteins, DNA.

### SPICA

The original Shinoda-DeVane-Klein (SDK) framework, developed in 2007 for modeling the self-assembly of aqueous surfactants (110, 111), served as the foundation for the SPICA CG potential. It was parameterized by fitting thermodynamic properties such as surface tension, density, and transfer free energy. SPICA effectively captures the self-assembly processes of surfactants and lipids with realistic interfacial behavior. Recently, its applications have extended beyond its initial scope to include simulations of lipids and proteins in a polar solution (112). The SPICA protein model incorporates an elastic network (EN) model to maintain the initial secondary and tertiary structure of proteins in solution during CG simulations (56). (113). Drawing from prior studies that combine the EN model to retain secondary and tertiary structures, as in the Martini model, the application of strong harmonic restraints (see Table 1 and Fig. 3) has been found to be necessary (114). Notably, both the cutoff distance and spring constant in the EN model can be adjusted to suit the target system. The application of the EN model on top of the SPICA model restricts the study of large conformational changes in structured proteins, such as protein unfolding driven by temperature or mechanical forces.

Nonetheless, SPICA has demonstrated the ability to accurately describe protein assemblies in viral capsids. It was applied to the poliovirus, an icosahedral capsid consisting of 60 copies each of four proteins (VP1, VP2, VP3, and VP4). The CG simulation, initialized from an equilibrated AA structure and incorporating the intrachain EN bonds, maintained a stable capsid over a 1-*μ*s simulation. The average capsid radius was 132.8 Å, closely aligning with the 133.6 Å observed in AA-MD (112). In the latest version of SPICA version 2 (97), several improvements were made to better model protein-lipid interactions and IDP systems. The previous version tends to over-stabilized protein adsorption on lipid membranes, causing soluble proteins to bind too tightly. To address this, the Lennard-Jones (LJ) parameters for backbone (BB)-lipid interactions were modified to better reproduce protein-binding sensitivity. SPICA 2 was tested on several proteins: lysozyme, phospholipase A2, the pleckstrin homology domain of PDK1, and the human micelle-bound *α*-synuclein (i.e., *α*-syn). Notably, *α*-syn chain remains in the helical form upon membrane binding.

For IDP systems, SPICA incorporates several key modifications to improve accuracy. These include removing the EN model, introducing secondary structure-dependent nonbonded interaction parameters for the protein backbone, and reoptimizing nonbonded parameters for all amino acids. These changes enable SPICA to model the radius of gyration of IDPs, the free energy of peptide association in water, and interactions between lipids and transmembrane proteins more effectively. The latest CG potential parameters can be obtained from the webpage (https://www.spica-ff.org/). Currently, SPICA does not support posttranslational modifications, and thus their effect on protein conformation remains elusive.

### GōMartini 3

To address the limitations of standard Martini 2 in capturing large conformational changes in proteins, the GōMartini approach was introduced as an extension of the Martini force field (54). In this approach, the EN typically used in Martini 2 simulations was replaced by Gō potentials mapped as LJ interactions between BB beads and parametrized as in Table 1 and Fig. 3. These LJ interactions were calculated based on the native contact map, which was derived using optimized CM determination strategies (115, 116). Despite several applications in protein folding (e.g., α-helices and β-hairpins), sampling of protein flexibility in the Man5B enzyme, and analysis of mechanical failure in self-assembling peptide fibrils (117), early models faced technical limitations in efficiency and scalability. These challenges motivated further developments, ultimately leading to the introduction of GōMartini 3.

The recent GōMartini 3 model (118), implemented on the Martini 3 framework (119), represents a significant advancement and addresses several limitations of its predecessor (54). In the previous implementation, Gō potentials between residue pairs were mapped as intramolecular interactions, the GōMartini 3 introduces virtual interaction sites placed on the BB beads (near the C*α* atoms). These virtual sites define the interactions between residue pairs using LJ potentials, enabling better computational efficiency through parallelization and the implementation of nonbonded cutoffs. Although the contact map remains unchanged, contacts are now defined within a BB–BB distance, ranging from 0.3 nm to 1.1 nm. This range ensures meaningful interactions: the 0.3 nm lower limit prevents artifacts from overlapping beads in dense regions, whereas the 1.1 nm upper limit aligns with Martini 3 nonbonded cutoff, excluding distant pairs unlikely to contribute to structural stability. Additionally, nonbonded interactions between BB beads are excluded for residue pairs defined via virtual sites. This exclusion improves the packing of protein structures and enhances their stability during simulations.

GōMartini 3 has been successfully applied to protein-ligand and protein-protein interactions, demonstrating both versatility and effectiveness. For example, it has been used to study the binding of benzene to L99A T4 lysozyme, revealing enhanced flexibility in the binding pockets compared with the EN model (118). The method also accurately reproduced the conformational flexibility and hydration levels around the zinc-binding site of copper-zinc superoxide dismutase, aligning well with results from QM/MM MD simulations. Additionally, it identified the destabilizing allosteric effect of the G93A mutation on the zinc-binding site (120) and probed the mechanical stability of several biomolecular systems (98, 121, 122), including SARS-CoV-2 variants in complex with a potent nanobody. Specific mutations were found to significantly influence the formation and rupture of native and nonnative contacts under high mechanical loads, impacting the mechanical stability of these complexes. Furthermore, the hyper-mechanostability of bone sialoprotein binding protein in complex with fibrinogen-*α* was explored, revealing rupture forces exceeding 2 nN and highlighting key residue interactions that resist mechanical stress (122). The CG potential is available via the server at https://github.com/Martini-Force-Field-Initiative/GoMartini.

### CABS

In addition to physics-based models, statistics-based protein CG models, such as the CABS potential, represent also an alternative in modeling large conformational changes in proteins. The CABS-fold were used in protein prediction competitions (e.g., CASP(57)) and their extension to capture flexibility (i.e., CABS-flex) in peptide-protein recognition and protein complex (123) enabling extensive rearrangements of the protein chain. These CG models rely on extensive experimental data, including folded protein structures deposited in the PDB, to derive potential energy functions. The functions are based on the observed frequency of structural features such as bond distances, angles, dihedral torsions, and residue-residue contacts. The CABS model excels in structure prediction of proteins and can even perform unassisted folding simulations with greater success compared with previous CG approaches. However, their performance is limited by a lack of transferability to systems outside their original parametrization, especially in interactions involving proteins and other biomolecules (see Table 1 and Fig. 3).

CABS was able to explore large conformational changes of peptide-protein recognition via CABS-dock (124). The p53-MDM2 complex, a key target in anticancer drug design, where experimental data suggest significant rearrangements in the flexible N-terminal region of MDM2. Due to its large size and flexibility, atomistic models struggle with exhaustive binding dynamics studies for this complex. Using CABS-dock, simulations successfully generated near-native models without prior knowledge of the p53 peptide structure or binding site, aligning well with experimental data and the role of the N-terminal domain in binding (37). More recently, CABS-dock was employed within an integrative modeling to predict the self-assembled structures of protein fibrils such as *β*-amyloid by incorporating structural restraints that mimic experimental conditions. Although such a protocol does not determine the overall structure of the assembled fibrils, and yet limits the whole exploration of the conformational space in proteins, it still yields an efficient method for protein structure determination(125). The CG potential can be accessed from the server http://biocomp.chem.uw.edu.pl/CABSflex2.

### UNRES

The UNRES potential was implemented for massively parallel CPU architectures (126). Although this allowed simulations of large protein systems, such as those with 150,000 residues, the computational cost remained high, often requiring several days of wall-clock time per trajectory, even with 24-core CPUs (127). Recently, the code was enhanced with hybrid parallelization using both MPI and OpenMP on GPUs, further improving performance (99). In a significant advancement, the UNRES CG simulation framework now supports GPU acceleration, representing a major step forward in computational efficiency. This update reduces dependence on large CPU clusters and dramatically speeds up simulations, aligning UNRES with other state-of-the-art CG frameworks that leverage GPU-based architectures (128). Additionally, UNRES potentials can incorporate experimental restraints from NMR, enabling the modeling of multistate systems and IDPs, including those with disordered regions (129). This capability is partly enabled by the model’s well-developed parametrization of protein flexibility (see Table 1 and Fig. 3). CG potentials for large DNA/RNA molecules are also parametrized under UNRES framework (130) A web-based implementation of the UNRES package is available at https://unres.pl.

### AWSEM

AWSEM CG potential (100) describes each amino acid using C*α*, C*β* and O atoms (except glycine) and is primarily physics-based approach, with a small knowledge-based term that biases local sequence of nine residues (or shorter) toward conformations seen in known protein structures. The potential uses a detailed backbone and one interaction site per side chain, incorporating an implicit solvent model that includes hydrophobic burial and explicit, water-mediated, nonadditive interactions (see Table 1 and Fig. 3). Originally implemented as AWSEM-MD in the LAMMPS molecular dynamics package using CPU, the model has been successfully applied to predict the structures of globular natural and designed *α*/*β* proteins, as well as polytopic membrane proteins (131). AWSEM-MD has also been used to study protein folding, association, and aggregation, and has demonstrated strong performance in CASP competitions, particularly when incorporating co-evolutionary and template-based information. A cross-compatible implementation of AWSEM for proteins, integrated with the 3SPN2 CG DNA model in the OpenMM framework, enabled the simulation of large-scale translocation in the bacteriophage T7 gp4 helicase–DNA complex (132). This study captured multiple intermediate conformational states and revealed transient DNA-protein and protein-protein interactions that facilitate long-range subunit translocation. Also, it has been used to extend the timescale of AA-MD for the study of the assembly of silk nanofibrils at the growth state (133). Notably, the GPU-accelerated OpenMM implementation (134) achieved a 30-fold speed-up compared with the original CPU-based LAMMPS version. The CG potential is openly available at https://github.com/npschafer/openawsem.

## ML for biomolecular backmapping

**Figure 4:**
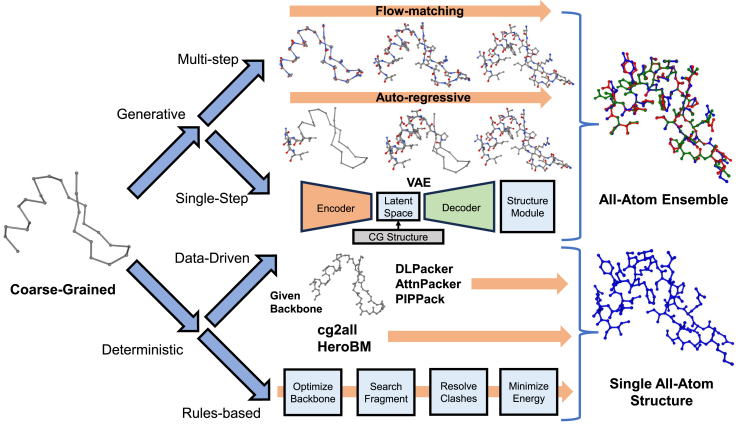
Methods for backmapping coarse-grained simulations to all-atom resolution. Traditional deterministic approaches (*bottom row*) follow a rule-based approach. Numerous data-driven methods have been developed to predict single structures from protein backbones or coarser representations. Recent generative approaches (*top row*) can predict an ensemble of all-atom configurations given a coarse-grained structure and random noise. Single-step predictions can be made by VAE or GAN-based models and generally are faster but less accurate. Structures with improved bond quality and fewer interresidue clashes can be generated in an iterative manner based on a flow-matching or diffusion objective, which may also be combined with autoregressive decoding.

Although coarse-graining has become an increasingly powerful tool for expanding time and length scales of molecular simulation, there are numerous scenarios in which it is desirable to restore AA resolution to CG trajectories in order to develop mechanistic understanding, compute observables contingent on atomistic degrees of freedom, or make comparisons against experimental measurements (135,136,137,138,139,140,141,142,143,144). Backmapping is a general term for the process of restoring degrees of freedom lost during the coarse-graining process (see Fig. 4). Early backmapping approaches typically follow sets of heuristics to produce a rule-based mapping from CG to AA. Initial structures are obtained either by searching fragment libraries (136, 145, 146) or by a geometrically guided scheme (55, 147,148,149,150,151). Structures are then refined to reduce steric clashes and unphysical bond angles and lengths, followed by optional energy minimization (147, 151). Although these approaches can be fast and produce reasonably stable AA structures, the optimization procedure can also produce unphysical dihedral angles or may fail to resolve spurious configurations such as “punched” aromatic rings (151). Furthermore, most rule-based methods are deterministic and are therefore unable to recover the ensemble of AA structures that correspond to the one-to-many backmapping of a single CG trace.

In the last decade, the development of generative deep-learning-based architectures such as generative adversarial network (GANs) (152) and variational auto-encoders (VAEs) (153) have enabled the generation of physically meaningful AA ensembles from CG conditioning and Gaussian noise. (154,155,156). Given backmapping is an inherently one-to-many prediction process, these generative techniques are more extensible than deterministic rule-based approaches. Li et al. (154) and Stieffenhofer et al. (157) leveraged GANs to backmap MD simulations of condensed-phase polymer systems and showed strong structural performance as well as transferability between phases. Wang et al. (158) developed a VAE approach to backmap small gas-phase molecules, and later (159), they enhanced their model using a graph neural network to backmap fast-folding proteins. Shmilovich et al. (156) developed a conditional VAE based on 3D voxels of consecutive MD frames to backmap temporally coherent AA trajectories. Although some chemical transferability in polymer melt systems was demonstrated by Stieffenhofer et al. (155), these earlier approaches tended to be system specific, which both necessitates substantial AA training data for the molecule of interest and requires the training of independent models for each molecular system of interest.

More generic chemical transferability, especially for machine learning-based approaches, has been achieved in the related field of protein side-chain packing (PSCP) (160,161,162,163,164). This task can be considered a specific type of backmapping, in which side-chain atoms are predicted given protein backbone atoms (C*α*, C, O, N) and primary sequence. Rule-based methods for PSCP such as SCWRL (165) and RosettaPacker (166) efficiently search rotamer libraries using energy-based optimization. Transferable deep-learning-based methods such as SIDEPro (160) were developed to learn improved energy functions given pairwise atom distances, and methods such as DLPacker and OPUS-ROTA4 (161, 163) predict side-chain positions given 3D voxel densities. McPartlon et al. demonstrated significant improvements with their AttnPacker (167) model, which combines a SE-3 transformer (168) and Tensor Field Network (169) to predict per-residue confidence scores and sequence (inverse-folding) in addition to side-chain configurations. Randolph et al. (170) incorporated invariant point message passing to achieve similar results at significantly improved computational efficiency. Although PSCP is posed very similarly to backmapping and packing models have inspired ML-based backmapping approaches, these methods are designed for static low-energy or de novo protein structures and require knowledge of all backbone atoms. Prediction of side-chain conformational ensembles has also been less of a focus for PSCP, and most ML-based methods are deterministic, but recent work by Zhang et al. (171) and the AlphaFold3 (172) structure module both incorporate diffusion-based approaches that are capable of producing conformational diversity.

Yang et al. (173) introduced the first chemically transferable C*α*-only backmapping model using a VAE architecture and demonstrated strong results when trained on IDPs from the Protein Ensemble Database (PED) (174). Han et al. (175) elaborated upon this work by employing a vector-quantized VAE and performing diffusion in the learned latent space. Jones et al. (50) developed an autoregressive diffusion model, called DiAMoNDBAck, that showed strong structural performance and conformational diversity when trained on the PED (174) and PDB (176) and demonstrated further improvements upon fine-tuning on molecular dynamics trajectories. Furthermore, DiAMoNDBAck outperformed previous generative models in reproducing conformational diversity present in experimental structures. Liu et al. (177) introduced a diffusion-based scheme with manifold constraints (i.e., guiding generation based on structural criteria) to enable transferability of a single backmapping model to various CG maps. Heo et al. (178) and Angioletti et al. (179) leveraged equivariant architectures to improve flexibility toward varied CG representations; however, these methods are deterministic and therefore cannot produce conformational diversity or sample multiple atomistic structures consistent with one CG configuration. Chennakesavalu et al. (180) used a transformer to map AA configurations from molecular simulations of amino acid tetramers onto C*α* backbones to better recapitulate statistically meaningful diversity. Recently, Jones et al. (181) introduced the FlowBack model, which uses a physically informed prior and flow-matching objective (182, 183) to efficiently generate accurate AA samples for CG conformations. A version of this model was trained to predict to backmap DNA-protein complexes as well, and the flow-matching concept is generalizable to any biomolecular system and CG mapping.

## Challenges and future perspectives

As foundational physically inspired MLPs expand their applicability to multiple (bio)molecular spaces, their design must be carefully optimized to ensure effective extrapolation to unknown conformations, which often arise in simulations of biological processes. The integration of active, transfer, and meta-learning algorithms into the training of these MLPs is an active area of research (184,185,186,187,188,189), offering a powerful means to efficiently explore vast conformational spaces and thereby facilitate compliance with the EAST requirements. The reduction of model parameters, resulting from improved training sample generation, along with efficient large-scale ML algorithms, such as those implemented in the JAX library (190), will improve inference time and enable QM-based biomolecular simulations on nano- to microsecond timescales. Another crucial factor in developing robust MLPs is incorporating physical models to accurately capture long-range interactions during their construction. Recent efforts have shown that explicitly incorporating physically inspired functional forms to treat these interactions in ML potential development can overcome the limitations of the locality assumption, a key feature for scalability and transferability, without increasing the complexity of molecular representations. Despite these advancements, a more in-depth understanding of how the choice of physical models affects the performance of MLPs remains lacking, particularly in complex simulations such as ligand binding in proteins or allosteric response. Alternative approaches, such as ML-augmented semiempirical models and hybrid MM/ML methods, also face challenges that require further investigation. Among these challenges are ensuring robust extrapolation to unrepresented electronic environments (e.g., charged systems, excited states) and optimizing their integration with existing computational frameworks (e.g., DFTB, FHI-aims, GROMACS, CP2K).

CG potentials have demonstrated their versatility in modeling large-scale biomolecular systems and capturing long-time dynamics approaching biological timescales. Many CG approaches, including those discussed in this work, impose restraints to maintain secondary and tertiary structures, which can limit their capacity to represent disordered or highly flexible conformational states. Capturing protein flexibility more accurately requires refined backbone representations. For example, SIRAH uses a three-bead backbone, which improves its capacity to model certain flexible structures, but not their interconversion. Such transitions between transient metastable states may be explored by combining the recently developed Martini 3-IDP model (191), optimized using atomistic simulations of various IDPs, with the GōMartini 3 approach. In contrast, SPICA uses a more simplified representation, which can limit its applicability to processes involving transitions between metastable states or dynamic folding pathways. The CABS model, in contrast, does not rely on parameters derived from native structures, which makes it particularly well suited for studying single IDP behavior. However, since it is based on statistical potentials extracted from the PDB, its applicability beyond proteins is limited. Similarly, knowledge-based CG potentials such as UNRES and AWSEM are also suitable for modeling IDPs, as they do not depend on a predefined native structure. Nonetheless, their use remains largely restricted to proteins due to the complexity involved in their potential parametrization. Efforts to improve CG models using ML approaches are actively being explored through approaches such as CGnets (46, 47) and Boltzmann Generators (49). Alternatively, CG models can be refined by integrating cryo-EM ensemble reweighting methods (192), which estimate probability distributions directly from cryo-EM density maps. Such distributions can then be used to optimize CG potentials by improving the agreement between simulated ensembles and experimental data. Moreover, the enhanced accuracy, flexibility, and efficiency of the discussed ONIOM approach (32) can support modern structural determination methods for biomacromolecules (e.g., Cryo-EM, MicroED), which can subsequently be used to fine-tune CG models.

Although tremendous progress has been made in generative and transferable backmapping in recent years, future work will enable extensibility or fine-tuning to arbitrary CG maps, chemical systems, and thermodynamic state points; incorporate physical priors to provide informative inductive biases; enforce the Boltzmann distribution in the generated conformational ensemble; and improve inference efficiencies to enable applications to ultralarge molecular systems and on-the-fly backmapping of biomolecular simulations. In the upcoming years, we expect the emergence of several ML methodologies that will integrate quantum-resolved structural and energetic property data with low-resolution CG methods through ML-assisted backmapping, bridging time and length scales in biomolecular simulations.

## Acknowledgments

A.B.P. acknowledges financial support from the National Science Center, Poland, under grant 2022/45/B/NZ1/02519 and gratefully acknowledges Polish high-performance computing infrastructure PLGrid (HPC Centers: ACK Cyfronet AGH) for providing computer facilities and support within computational grant no. PLG/2023/016519 and no. PLG/2024/017332. This material is based on work supported by the 10.13039/100000001National Science Foundation under grant no. CHE-2152521 (A.L.F.). A.B.P. and L.M.S. thank CECAM for financially supporting the organization of the workshop “Leveraging Machine Learning for Sampling Rare Events in Biomolecular Systems,” where the discussions leading to this work began. A.H.C. and L.M.S. are also grateful to the Research Experience for Peruvian Undergraduates (REPU) program for its organizational support.

## Author contributions

All authors contributed equally to the conceptualization, writing, and review of the manuscript.

## Declaration of interests

A.L.F. is a cofounder and consultant of Evozyne, Inc., and a coauthor of US Patent Applications 16/887,710 and 17/642,582, US Provisional Patent Applications 62/853,919, 62/900,420, 63/314,898, 63/479,378, 63/521,617, and 63/669,836, and International Patent Applications PCT/US2020/035206, PCT/US2020/050466, and PCT/US24/10805.
